# Bacterial Microbiota and Metabolic Character of Traditional Sour Cream and Butter in Buryatia, Russia

**DOI:** 10.3389/fmicb.2018.02496

**Published:** 2018-10-22

**Authors:** Jie Yu, Lanxin Mo, Lin Pan, Caiqing Yao, Dongyan Ren, Xiaona An, Tsedensodnom Tsogtgerel, Heping Zhang, Wenjun Liu

**Affiliations:** Key Laboratory of Dairy Biotechnology and Engineering, Ministry of Education, Key Laboratory of Dairy Products Processing, Ministry of Agriculture, Inner Mongolia Agricultural University, Huhhot, China

**Keywords:** Buryatia, sour cream, butter, bacterial microbiota, metabolic character

## Abstract

Traditional sour cream and butter are widely popular fermented dairy products in Russia for their flavor and nutrition, and contain rich microbial biodiversity, particularly in terms of lactic acid bacteria (LAB). However, few studies have described the microbial communities and metabolic character of traditional sour cream and butter. The objective of this study was to determine the bacterial microbiota and metabolic character of eight samples collected from herdsmen in Buryatia, Russia. Using single-molecule real-time (SMRT) sequencing techniques, we identified a total of 294 species and/or subspecies in 169 bacterial genera, belonging to 14 phyla. The dominant phylum was Firmicutes (81.47%) and the dominant genus was *Lactococcus* (59.28%). There were differences between the bacterial compositions of the sour cream and butter samples. The relative abundances of *Lactococcus lactis*, *Lactococcus raffinolactis*, and *Acetobacter cibinongensis* were significantly higher in sour cream than in butter, and the abundance of *Streptococcus* *thermophilus* was significantly lower in sour cream than in butter. Using a pure culture method, 48 strains were isolated and identified to represent seven genera and 15 species and/or subspecies. Among these isolates, *Lactococccus lactis* subsp. *lactis* (22.50%) was the dominant LAB species. Ultra-performance liquid chromatography–quadrupole–time of flight mass spectrometry at elevated energy was used in combination with statistical methods to detect metabolite differences between traditional sour cream and butter. A total of 27,822 metabolites were detected in all samples, and Lys-Lys, isohexanal, palmitic acid, Leu-Val, and 2′-deoxycytidine were the most dominant metabolites found in all samples. In addition, 27 significantly different metabolites were detected between the sour cream and butter samples, including short peptides, organic acids, and amino acids. Based on correlation analyses between the most prevalent bacterial species and the main metabolites in sour cream, we conclude that there may be a connection between the dominant LAB species and these metabolites. This study combined omics techniques to analyze the bacterial diversity and metabolic character of traditional sour cream and butter, and we hope that our findings will enrich species resource libraries and provide valuable resources for further research on dairy product flavor.

## Introduction

Russian sour cream, a widely popular fermented cream product, plays an important role in the diet of Russian people. Sour cream has a milky white color, fragrant flavor, and delicious taste. It can be eaten directly with bread, and is widely used as a condiment, in cooking, and baking various types of pastry. Russia consists of 193 different ethnic groups and indigenous peoples, and each region has its own unique culture and customs, as well as its own types and methods of preparing dairy products. The processing technique used to make sour cream (*susegei*) in Buryatia, Russia is simple: raw milk is stored in casks in a dark and dry room for 3–4 days. Natural fermentation leads to layering of the fermented milk, with the top layer being sour cream. The addition of hot water and stirring are performed to make butter (*tohon*), which is then stored in a sheep stomach. Butter has been consumed for 1000s of years in Buryatia. Obviously, many natural microorganisms originating from the raw milk and environment are involved in these fermentation processes. Therefore, a comprehensive and systematic analysis of the complex bacterial communities within sour cream and butter in Russia would be useful in the commercialization of these products.

In previous studies, traditional culture methods have been used to investigate the microbial communities in traditionally fermented dairy products (Mathara et al., 2004; Elionora Hantsis-Zacharov, 2007; Giannino et al., 2009). However, microbial communities described solely on the basis of culturing are often incomplete, as many species are unculturable or poorly represented by the culturing process (Duniere et al., 2017). With the development of polymerase chain reaction (PCR) technology, alternative culture-independent techniques have facilitated investigations of the complex microbial microbiota in traditional dairy products. Among these, metagenomic sequencing of microbial DNA is an important culture-independent method that could provide a comprehensive microbiota profile independent of phenotypic traits and the cultivability of individual microbes. This modern molecular approach has been used to identify the microbiota of different types of traditional fermented dairy products, such as Irish artisanal cheese (Quigley et al., 2012), Italian Plaisentif cheese (Dalmasso et al., 2016), Belgian Herve cheese (Delcenserie et al., 2014), Mongolian naturally fermented cow’s milk (Liu et al., 2015), and Xinjiang yogurts (Xu et al., 2015). Recently, a new high-throughput sequencing tool, the third-generation Pacific Biosciences (Menlo Park, CA, United States) single-molecule real-time (SMRT) sequencing technology, was developed to generate long reads and allow high taxonomic resolution at the species level when coupled with full-length 16S ribosomal RNA (rRNA) gene sequencing (Mosher et al., 2014). This technique has been successfully applied to evaluate infant formula safety (Zheng et al., 2016) and to investigate bacterial profiles in traditional fermented koumiss (Gesudu et al., 2016) and artisanal Kazakhstan cheese (Li et al., 2017). To our knowledge, microbiota studies have focused on cheeses and fermented milks; no simultaneous studies of the microbial communities of traditional sour cream and butter have been conducted.

Given the wide popularity of Russian traditional sour cream and butter, and that the Buryatia region maintains traditional dairy processing methods, we collected samples of traditional sour cream and butter from local households in Buryatia, Russia. We first used SMRT sequencing technology to assess the bacterial population present in sour cream and butter samples, and tested for differences in bacterial composition between sour cream and butter. We then isolated lactic acid bacteria (LAB) using culture methods with the aim of preserving these valuable LAB resources for subsequent research and industrial applications. Third, to further understand the nutritional characteristics of sour cream and butter, we conducted ultra-performance liquid chromatography–quadrupole–time of flight (UPLC–Q–TOF) mass spectrometry at elevated energy (MS^E^) to detect the metabolites of sour cream and butter.

## Materials and Methods

### Sample Collection

A total of five traditional sour cream (BL9, BL11, BL17, BL21, and BL26) and three butter (BL14, BL18, and BL20) samples were collected from six different regions in Buryatia, Russia. The pH values of these samples ranged from 4.51 to 5.03. Samples were collected in sterilized containers and transported to the laboratory in a 4°C vehicle-mounted refrigerator.

### Isolation and Identification of LAB

Lactic acid bacteria strains were isolated from the eight samples using a previously described culture method (Yu et al., 2015). A total of 48 Gram-positive and catalase-negative isolates were obtained from the samples. Isolates were preserved in milk containing 0.1% sodium glutamate and stored at −40°C.

We identified the 48 isolates using 16S rRNA gene sequencing technology. The 16S rRNA gene primers used were 16S-FA (GCAGAGTTCTCGGAGTCACGAAG AGTTTGATCCTGGCTCAG) and 16S-RA (AGCGGATCACTTCACACAGGACTA CGGCTACCTTGTTACGA). Genomic DNA extraction and PCR amplification were performed as previously described (Yu et al., 2011). DNA sequencing was performed by Majorbio Bio-Pharm Technology Corporation Limited (Shanghai, China). A phylogenetic tree was constructed based on the generated sequences using MEGA v. 7.0 software^1^.

### PacBio SMRT Sequencing and Bioinformatics Analysis

We centrifuged 3 *g* sour cream and butter samples at 10,000 × *g* for 20 min in an Eppendorf 5810R centrifuge. The supernatant was then removed and the cream attached to the tube was removed using sterile swabs. The remaining pellet was re-suspended in 500 mL of sterile water; 500 mg of lysozyme was added prior to incubation for 12 h at 37°C to maximize bacterial DNA extraction. Genomic DNA was extracted using the DNeasy Mericon Food Kit (69514, Qiagen, Hilden, Germany) according to the manufacturer’s instructions. DNA concentration and purity were evaluated using 0.8% agarose gel electrophoresis and optical density using a NanoDrop ND-1000 spectrophotometer (Thermo Fisher Scientific, Wilmington, DE, United States).

Genomic DNA was used as a template for PCR amplification of the full-length 16S rRNA gene for SMRT sequencing. The primers and reaction conditions were performed as described previously (Hou et al., 2015). The quality of the PCR products was verified using the Agilent DNA 1000 Kit and an Agilent 2100 Bioanalyser (Agilent Technologies, Santa Clara, CA, United States). Purified PCR products were used to construct DNA libraries with the Pacific Biosciences Template Prep Kit 2.0. The P6–C4 chemistry of the PacBio RS II instrument (Pacific Biosciences) was used to sequence the purified amplification products.

Raw sequence data were generated using the RS_ReadsOfinsert.1 protocol available on the SMRT Portal (v. 2.7) (Hou et al., 2015). The Quantitative Insights into Microbial Ecology (QIIME) package (v. 1.7) was used to extract high-quality sequences, and these sequences were then aligned under 100% sequence identity clustering to obtain representative sequences using the PyNAST and UCLUST systems (Caporaso et al., 2010; Edgar, 2010). Sequences were subsequently classified into operational taxonomic units (OTUs) below a threshold of 97% using UCLUST software (Lozupone et al., 2006). The Ribosomal Database Project II database was used to assign the taxonomy of each OTU-representative sequence at an 80% confidence threshold (Cole et al., 2007). A *de novo* taxonomic tree was constructed using a representative OTU set in the FastTree software for downstream analysis (Price et al., 2009). The α-diversity was evaluated using Shannon–Wiener, Simpson’s diversity, Chao1, and rarefaction estimators. R programming language v. 3.1.2^2^ and Origin v. 8.5 software were used to test correlations and produce graphs.

### Determination of Metabolites by UPLC–Q–TOF MS^E^

The dairy products were centrifuged at 4000 × *g* for 10 min, and 1 mL of suspension was collected after the fat layer was discarded. We added 7 mL of acetonitrile solution to the suspension, which was then vortexed for 2 min before high-speed centrifugation at 12,000 × *g*. The supernatant was concentrated in a vacuum concentrator, reconstituted in 500 μL of 40% (v/v) acetonitrile solution, filtered through a 0.22-μm water-insoluble microporous membrane, and stored at −20°C. An ACQUITY UPLC/Xevo G2 Q TOF–MSE chromatography system (Waters Corp., Milford, MA, United States) was used to extract MS spectral peaks of dairy products; it was equipped with an electrospray ionization (ESI) source that operated in both positive and negative ion modes (ESI+ and ESI−). Pre-treated milk samples were chromatographed using a C18-UPLC system. The column was HSS T3 (2.1 mm × 100 mm × 1.8 μm, Waters Corp.), the flow rate was 0.45 mL/min, and the column temperature was 40°C. The ion scan mode, liquid phase, mass spectrometry conditions, and sample volume settings were selected according to a previous study (Pan et al., 2018). Raw data obtained by UPLC-Q-TOF MS^E^ were collected in continuum mode and processed using the MassLynx and Progenesis QI software (Waters Corp.). Data preprocessing procedures including peak alignment, peak identification, and deconvolution were completed, and the resulting data set was imported into EZinfo software. PCA and orthogonal projections to latent structures discriminant analysis (OPLS-DA) were then performed on the data set. Significant differences in metabolites between dairy products were determined when VIP ≥ 1, fold change ≥ 2, and *P* < 0.05. The selected differential metabolites were identified using the Human Metabolome Database (HMDB^3^), Kyoto Encyclopedia of Genes and Genomes (KEGG) ^4^, ChemSpider ^5^, and METLIN^6^ databases.

### Nucleotide Sequence Accession Number

All PacBio SMRT sequencing data reported in this study were deposited at Metagenomic Rapid Annotations using the Subsystems Technology database (accession no. mgp83644)^7^. All 16S rDNA sequences from LAB isolates were submitted to the National Center for Biotechnology Information database (accession nos. MF784091–MF784111 and MF784124–MF784150).

## Results

### Enumeration and Identification of LAB

**FIGURE 1:**
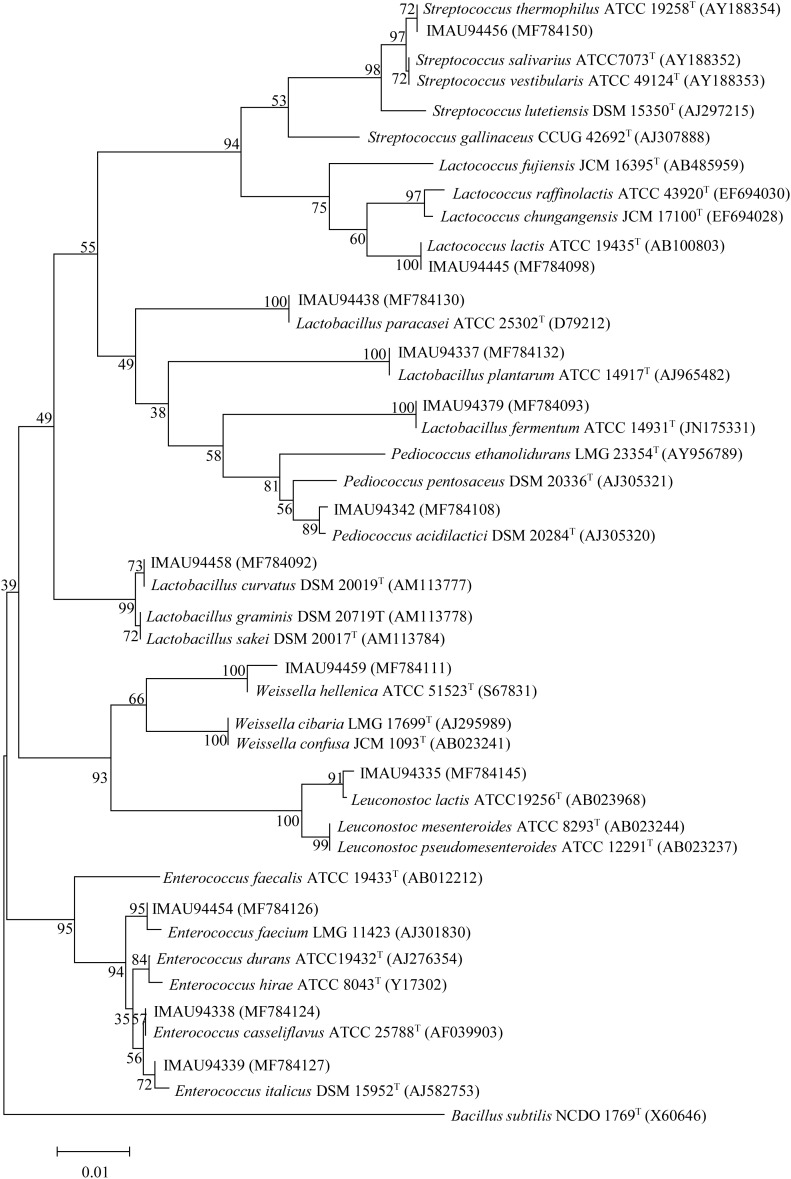
Neighbor-joining tree showing the phylogenetic relationships between isolates and the type strains of related genera based on 16S rRNA gene sequences. *Bacillus subtilis* was used as an outgroup.

Viable counts of LAB present in the eight dairy samples of butter and sour cream ranged from 5.19 ± 0.01 to 8.37 ± 0.01 l g CFU/g. We obtained 48 isolates by pure culture, which were presumptively identified as LAB based on Gram-positive and catalase-negative phenotypes. Partial 16S rRNA gene sequencing was further used to identify these isolates belonging to seven genera and 15 species, including *Enterococcus* *casseliflavus* (2 strains), *E*. *italicus* (2 strains), *E*. *faecium* (2 strains), *Lactobacillus fermentum* (2 strains), *Lactobacillus paracasei* (1 strain), *Lactobacillus plantarum* (7 strains), *Lactobacillus curvatus* (1 strain), *Lactococcus lactis* (16 strains), *Lactococcus garvieae* (2 strains), *Leuconostoc mesenteroides* (3 strains), *Leuconostoc garlicum* (2 strains), *Leuconostoc lactis* (1 strain), *Pediococcus* *acidilactici* (2 strains), *Streptococcus* *thermophilus* (4 strains), and *Weissella hellenica* (1 strain) (Figure 1).

### Bacterial Community Diversity and Richness in Butter and Sour Cream

**FIGURE 2:**
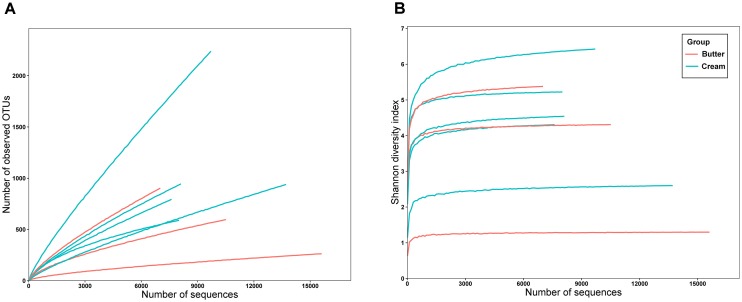
Rarefaction analysis **(A)** and Shannon diversity **(B)** estimates of the SMRT sequencing reads of bacteria in sour cream and butter samples.

A total of 81,730 high-quality SMRT sequencing reads (average: 10,216 reads/sample, range: 7,034–15,783, standard deviation [SD]: 2,875) were obtained from the eight samples. Following alignment and sequence identity clustering, 7,301 OTUs were generated with an average of 912.63 representative OTUs (SD: 551.18) for each individual sample. The number of reads, observed species (155.34–1689.80), Chao1 index (484.77–13724.40), Simpson’s index (0.267–0.909), and Shannon–Weiner index (1.275–6.314) of the eight samples are shown in Table 1. As shown in Figure 2, the Shannon diversity and rarefaction curves suggest that the overall bacterial phenotypes were well captured at the current sequence depth.

**Table 1:** Operational taxonomic units (OTUs) number of sequence and OTUs, microbial diversity and richness indexes of sour cream and butter samples.

Sample type	Sample name	No. of Reads	No. of OTU	Chao1 index	Shannon index	Simpson index	Observed species
Butter	BL14	7034	903	5253.110	5.378	0.909	902.700
	BL18	10595	598	1390.210	4.277	0.823	453.480
	BL20	15783	265	484.769	1.275	0.267	155.340
Sour cream	BL9	8396	949	3605.430	4.518	0.800	841.800
	BL11	8168	799	3973.450	4.292	0.743	744.200
	BL17	8148	592	1134.260	5.215	0.898	548.140
	BL21	13808	943	2856.540	2.532	0.454	544.320
	BL26	9798	2252	13724.40	6.314	0.886	1689.80
	Mean ± SD	10216.25 ± 2874.91	912.63 ± 551.18	4052.77 ± 3952.15	4.23 ± 1.51	0.72 ± 0.22	734.97 ± 423.94

### Bacterial Composition of Butter and Sour Cream

**FIGURE 3:**
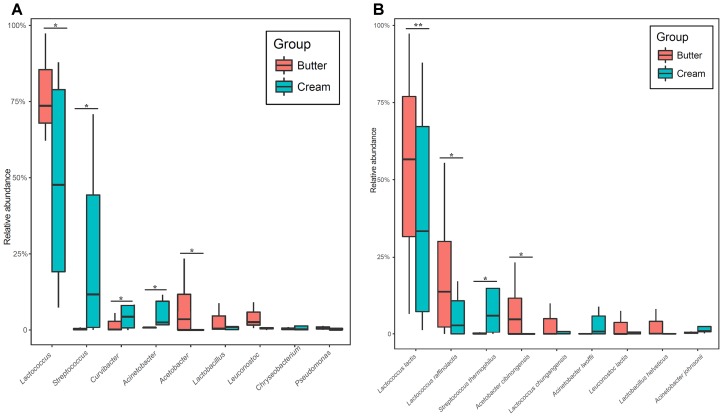
Boxplots of the relative abundance of high abundance (>1%) bacteria detected in sour cream and butter samples at the genera **(A)** and species level **(B)** with significant differences.

We identified nine bacterial phyla in butter samples and 14 in sour cream samples. The major bacterial phyla (with average relative abundance > 1%) in all samples were Firmicutes (81.47%), Proteobacteria (16.11%), and Bacteroidetes (2.05%); there were no significant differences in bacterial relative abundance between butter and sour cream samples. At the genus level, 75 and 155 bacterial genera were detected in butter and sour cream, respectively. *Lactococcus* (77.73%), *Acetobacter* (7.80%), *Leuconostoc* (4.10%), *Lactobacillus* (3.09%), and *Curvibacter* (1.92%) were the dominant genera in butter samples, whereas *Lactococcus* (48.21%), *Streptococcus* (23.63%), *Curvibacter* (8.15%), *Acinetobacter* (5.43%), *Lactobacillus* (2.21%), *Chryseobacterium* (1.90%), *Pseudomonas* (1.71%), and *Carnobacterium* (1.13%) were the dominant genera in sour cream samples. The relative abundances of *Lactococcus* and *Acetobacter* were significantly higher in sour cream than in butter, and those of *Streptococcus*, *Curvibacter*, and *Acinetobacteras* were significantly lower in sour cream than in butter (Figure 3A). At the species level, a total of 110 and 267 different bacterial species were identified in butter and sour cream, respectively (Supplementary Table S1). In butter samples, the most abundant species (with average relative abundance > 1%) were *Lactococcus lactis* (53.48%), *Lactococcus raffinolactis* (20.00%), *Acetobacter cibinongensis* (7.72%), *Lactococcus chungangensis* (3.30%), *Lactobacillus helveticus* (2.72%), *Leuconostoc* *lactis* (2.48%), and *Leuconostoc mesenteroides* (1.43%). The most abundant species (with average relative abundance > 1%) in sour cream samples were *Lactococcus lactis* (39.39%), *S*. *thermophilus* (10.60%), *Lactococcus raffinolactis* (6.11%), *Acinetobacter lwoffii* (3.07%), *Lactococcus chungangensis* (1.76%), *Acetobacter cibinongensis* (7.72%), and *Acinetobacter johnsonii* (1.38%). The relative abundances of *Lactococcus lactis*, *Lactococcus raffinolactis*, and *Acetobacter cibinongensis* were significantly higher in sour cream than in butter, and those of *S*. *thermophilus* were significantly lower in sour cream than in butter (Figure 3B).

### Comparison of the Bacterial Community Structures of Butter and Sour Cream

**FIGURE 4:**
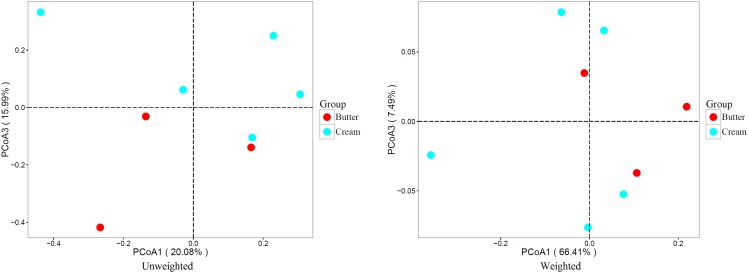
Weighted UniFrac principal coordinate analysis (PCoA) of the bacterial communities in butter and sour cream samples. Each symbol represents the butter and sour cream microbiota of one sample; butter and sour cream sample is represented by the respective color.

To compare the bacterial communities of butter and sour cream, we performed weighted and unweighted UniFrac principal co-ordinate analysis (PCoA) based on the OTU abundance table. As shown in Figure 4, both unweighted (principal components PC1 and PC3 accounted for 20.08 and 15.99% of the total variance, respectively) and weighted (PC1 and PC3 accounted for 66.41 and 7.49% of the total variance, respectively) UniFrac distances revealed apparent structural differences between the bacterial communities in the butter and sour cream samples.

### Metabolite Profiling of Butter and Sour Cream Samples

**FIGURE 5:**
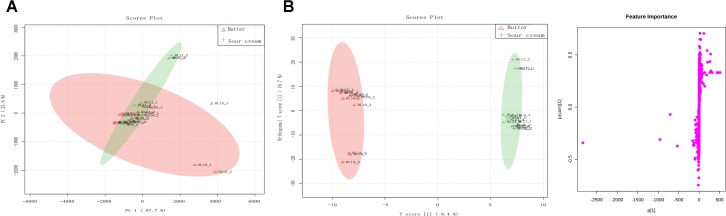
**(A)** Principal component analysis (PCA) score plots of the sour cream and butter metabolome. Color version available online. **(B)** Orthogonal partial least squares discriminant analysis (OPLS-DA): S-plots of the data for sour cream and butter samples.

A total of 27,822 metabolites (1,182 and 26,640 identified by positive and negative ion mode, respectively) were detected in all eight samples. Among these, Lys-Lys, isohexanal, palmitic acid, Leu-Val, and 2′-deoxycytidine were the most dominant metabolites in all samples. To visualize the metabolic differences between sour cream and butter, an unsupervised PCA was performed on the metabolome (Figure 5A). Principal components 1 and 2 represented 67.7 and 23.6% of the total variance, respectively. In the PCA score plots, symbols representing triplicate samples of sour cream and butter were not clearly separated, indicating the presence of many similar metabolites in the cream and butter samples. These data were further processed with a supervised OPLS-DA to identify differential metabolites. The score plot and S-plots are shown in Figure 5B, which demonstrates a clear clustered pattern between the two groups of samples. All *R*^2^*Y* and *Q*^2^ values were greater than 0.75, indicating high fitness and prediction ability. In the S-plot, each point represents a variable; a greater distance between a point and the origin indicates a greater contribution by that variable to the group difference, as well as greater confidence. According to the screening principle, the condition-matched metabolic differential variables were further analyzed and identified; finally, 27 significantly different metabolites were detected between the sour cream and butter samples (Table 2). Among these, the abundances of piperidine, Gly-Ser, Asn-Tyr, threonine, *N*-acetyl-L-leucine, Gln-Thr, Ser-Asn, tartaric acid, threonine, His-Ala, Pro-Asn, purine, butyric acid, and hexanal were higher in butter than in sour cream, whereas those of Met-Tyr, His-Asn, L-xylulose, decanoic acid, cystine, L-cysteine, L-lysine, Lys-Val, Tyr-Cys, L-alanine, uridine, aspartic acid, serine, and Gly-Ser-Pro-Met-Phe-Ala-Val were greater in sour cream than in butter.

**Table 2:** Significantly differential metabolites between sour cream and butter samples.

RT	M/Z	Identity of differential metabolites	Molecular formula	Molecular weight (Da)	Fold-change
7.2721	635.3858	gly-ser-pro-met-phe-ala-val	C_29_H_45_N_7_O_7_S	635.7750	0.0005
6.2968	89.0540	L-alanine	C_3_H_7_NO_2_	89.0930	0.0010
5.1123	269.1236	his-asn	C_10_H_15_N_5_O_4_	269.2570	0.0033
1.4752	240.1018	Cystine	C_6_H_12_N_2_O_4_S_2_	240.3005	0.0158
5.1591	312.1913	met-tyr	C_14_H_20_N_2_O_4_S	312.3850	0.0620
6.3737	245.1136	Lys-val	C_11_H_23_N_3_O_3_	245.3190	0.0824
5.8707	133.0824	Aspartic acid	C4H7NO_4_	133.1027	0.1007
8.1102	172.0855	Decanoic acid	C_10_H_20_O_2_	172.265	0.1182
4.4586	105.0292	Serine	C_3_H_7_NO_3_	105.0926	0.1719
1.4497	150.0533	L-xylulose	C_5_H_10_O_5_	150.1300	0.1829
5.6630	284.1233	tyr-cys	C_12_H_16_N_2_O_4_S	284.3310	0.2205
2.6256	121.0253	L-cysteine	C_3_H_7_NO_2_S	121.1580	0.2451
5.8045	244.1284	Uridine	C_9_H_12_N_2_O_6_	244.2010	0.2571
2.6700	146.0581	L-lysine	C_6_H_14_N_2_O_2_	146.188	0.2656
14.6074	88.0701	Butyric acid	C_4_H_8_O_2_	88.1050	2.5255
1.6908	100.0710	Hexanal	C_6_H_12_O	100.1590	2.7060
6.7740	120.0772	Purine	C_5_H_4_N_4_	120.1120	2.7497
16.1911	119.0818	Threonine	C_4_H_9_NO_3_	119.1192	2.9109
1.9344	226.1910	His-ala	C_9_H_14_N_4_O_3_	226.2320	4.8151
8.3437	229.1532	pro-asn	C_9_H_15_N_3_O_4_	229.2330	5.1604
14.2835	150.1104	Tartaric acid	C_4_H_6_O_6_	150.0868	5.9013
9.4422	247.1445	gln-thr	C_9_H_17_N_3_O_5_	247.2480	15.1180
9.4265	219.1488	Ser-asn	C_7_H_13_N_3_O_5_	219.1950	16.4850
5.8235	173.1270	*N*-Acetyl-L-leucine	C_8_H_15_NO_3_	173.2100	22.7870
2.2355	162.0737	Gly-ser	C_5_H_10_N_2_O_4_	162.1440	46.2500
4.0346	295.1642	asn-tyr	C_13_H_17_N_3_O_5_	295.2910	50.7320
2.3423	85.0050	Piperidine	C_5_H_11_N	85.1470	59.0590

### Correlation Between the Relative Abundance of Dominant Bacteria and Metabolites in Sour Cream

**FIGURE 6:**
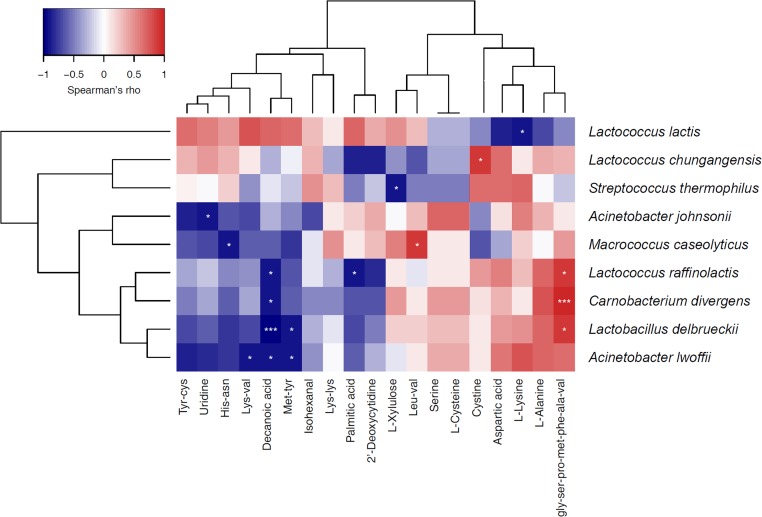
Heatmap showing the correlation relationships between the most prevalent bacterial genera (with average relative abundance of > 0.5%) and main metabolites in sour cream samples.

To investigate the relationship between the dominant bacteria and metabolites in the sour cream samples, we performed a correlation analysis between the most prevalent bacterial species (average relative abundance > 0.5%) and 19 main metabolites in sour cream. Because there were fewer butter samples, it was difficult to calculate the correlation between the bacterial species and metabolites in these samples. As shown in Figure 6, the predominant species *L*. *lactis* was positively correlated with the 12 metabolites that were of higher relative abundance, including decanoic acid, palmitic acid, L-xylulose, isohexanal, and some amino acids, and significantly negatively correlated with L-lysine. The sub-dominant species *S*. *thermophilus* was positively correlated with cystine, aspartic acid, uridine L-lysine, Lys-Lys, Tyr-Cys, and His-Asn, and significantly negatively correlated with L-xylulose. *L*. *chungangensis* was significantly positively correlated with cysteine, and *A*. *johnsonii* was significantly negatively correlated with uridine. *L*. *raffinolactis*, *L*. *delbrueckii*, and *Carnobacterium divergens* were significantly positively correlated with the peptide Gly-Ser-Pro-Met-Phe-Ala-Val, and significantly negatively correlated with decanoic acid. *A*. *lwoffii* was significantly negatively correlated with decanoic acid, Lys-Val, and Met-Tyr. *Macrococcus caseolyticus* was significantly positively correlated with Leu-Val, and negatively correlated with His-Asn. The results indicate that some of the metabolites in sour cream were the products of fermentation by the species with which they were correlated.

## Discussion

Traditionally fermented dairy products offer unique flavor and nutrition, which are closely related to the bacterial composition and milk resource. Therefore, the objective of this study was to combine omics techniques to analyze the bacterial diversity and metabolic character of traditional sour cream and butter collected in Buryatia, Russia. A total of 48 LAB strains were isolated and identified as seven genera and 15 species. A greater number of strains were identified as *Lactococcus lactis*, *Lactobacillus plantarum*, and *S*. *thermophilus* than as other species. SMRT sequencing results also indicated that *Lactococcus lactis* and *S*. *thermophilus* were the most abundant species in the sour cream and butter samples (Figure 3 and Supplementary Table S1). This result is similar to that of a previous study on traditional sour cream, which reported *Leuconostoc mesenteroides*, *Lactococcus lactis*, *Lactobacillus plantarum*, and *Lactobacillus helveticus* strains as the dominant isolates in sour cream from Kalmykiya and Tuva, respectively (Yu et al., 2015). In addition, all 15 isolated species were detected by SMRT sequencing, but these accounted for only 5.1% (15/294 species) of all species detected by SMRT sequencing. It is known that approximately 99% of microorganisms in the natural environment are not readily cultivable by traditional culture methods (Amann et al., 1995). Although the PacBio SMRT platform is indispensable for the systematic investigation of bacterial diversity and community structure in environmental samples, it can be coupled with full-length 16S rRNA sequencing to determine bacterial profiles at the species level. Using the SMRT sequencing data, we could improve medium components and culture conditions to isolate the desirable strains in a future study.

The 16S rRNA profiling of the microbiota found slight variations in the microbial compositions of the sour cream and butter samples. The relative abundances of *Lactococcus lactis* and *S*. *thermophilus* were significantly different (*P* < 0.05) between the butter and sour cream samples, and there were no significant differences among those of the other species between sample types. These samples were all prepared from cow’s milk, and the sampling region was more isolated; we speculate that these conditions led to the similarities in indigenous microflora between sample types. Liu et al. (2015) investigated microbial populations in naturally fermented cow milk samples from the Russian republics of Kalmykia and Chita using ribosomal-gene-targeted 454 pyrosequencing. *Lactobacillus* and *Lactococcus* were the predominant genera in samples from both Kalmykia and Chita in the present study. However, *Lactococcus* (51.46%) and *Streptococcus* (17.81%) have been reported to be the most dominant bacterial species in traditional Buryatian cottage cheese (Jin et al., 2018). The results of that study indicated that raw milk, external environment, processing method, and geographic location attributed to differences in microbial composition among traditional dairy products. As in our study, previous reports have confirmed that the exposure of traditional dairy products to different external environments during the manufacturing process impacted the microbiota composition of the final products (Feurer et al., 2004; Quigley et al., 2012). Furthermore, the abundances of lactococci, such as species of *Lactococcus*, *Leuconostoc*, and *Streptococcus*, were higher than lactobacilli in sour cream and butter in the present study. We conclude that lactococci may be able to better adapt to high-fat environments than lactobacilli. Although LAB have a low ability to hydrolyze fat, they could survive in sour cream and butter. Therefore, these strains should be further screened as starting cultures for the industrial production of sour cream.

*Streptococcus thermophilus* and *Lactococcus lactis* are recognized as the most important microorganisms in the dairy industry, and they commonly isolated from traditional fermented dairy products. It has been reported that *Lactococcus raffinolactis* coupled with *Lactococcus lactis* could be used as a new starter culture in fermented milk due to their strong complementarity during the fermentation process (Kimotonira et al., 2012). However, it is worth noting that low relative abundances were detected for certain taxa in the present study, including the genera *Acetobacter*, *Pseudomonas*, and *Chryseobacterium*, and species *Acinetobacter lwoffii*, *Acinetobacter johnsonii*, *Carnobacterium divergens*, and *Macrococcus caseolyticus*. *Acetobacter* can oxidize alcohol or sugars incompletely to form acetic acid, which is widely found in traditional fermented foods (Ishida et al., 2005; Kiryu et al., 2012). Members of the genus *Pseudomonas* demonstrate broad metabolic diversity and consequently are able to colonize a wide range of niches. *Pseudomonas* is a dominant active bacterial contributor to spoilage under aerobic conditions, even at refrigeration temperatures (Ercolini et al., 2011), and some species are opportunistic human and plant pathogens (Palleroni, 2010). Some cold-tolerant *Chryseobacterium* species, including *C*. *oranimense*, *C*. *haifense*, and *C*. *bovis*, have been detected in raw milk in Israel (Hantsis-Zacharov and Halpern, 2007). *Acinetobacter lwoffii* and *Acinetobacter johnsonii* commonly occur in marine fish and water, and are regarded as opportunistic fish pathogens. *Carnobacterium divergens*, a psychrotrophic and microaerophilic but oxygen-tolerating bacterium (Leisner et al., 2007), is predominant in industrial foods and is frequently associated with the spoilage of refrigerated meat and fish products (Barakat et al., 2000). These potential pathogens may cross-contaminate via several pathways, including animal bodies, feces, and processing environments. We therefore suggest that herdsmen be vigilant with respect to sanitary conditions during dairy production.

It is well known that cow milk contains, on average, 87.3% water, 3.4% protein, 3.6% fat, and 4.6% lactose per 100 g (Guy and Fenaille, 2006). However, Boudonck et al. (2009) found that cow milk contains a complex mixture of metabolites, and they identified 223 metabolites by liquid and gas chromatography/mass spectrometry methods, including amino acids, lipids, carbohydrates, nucleotides, energy metabolites, vitamins, cofactors, and short peptides. Fermentation is a metabolic process leading to the release of different chemical substances as metabolites, and these metabolites result from the activity of the microorganisms present within the food, the concentration of which changes over time from the manufacture of a fermented food to its consumption. Therefore, more studies are now focusing on the detection of the metabolic fingerprints and bacterial communities of fermented foods to evaluate their quality (Lee et al., 2015; Young et al., 2016). To date, reports regarding the identification of metabolites in traditionally fermented sour cream and butter are limited. The current study detected 27,822 metabolites, and significant amounts of Lys-Lys, isohexanal, palmitic acid, Leu-Val, and 2′-deoxycytidine were detected in the Russian sour cream and butter samples. Palmitic acid, the most common saturated fatty acid, is naturally present in butter, cheese, milk, and meat, as well as cocoa butter, soybean oil, and sunflower oil. The World Health Organization indicated that consumption of palmitic acid increases the risk of developing cardiovascular disease and may increase LDL levels in the blood. Furthermore, it has been reported that palmitic acid has the strongest effect in boosting the metastatic potential of CD36+ metastasis-initiating cells (Pascual et al., 2016). In addition, 27 significantly different metabolites were detected between the sour cream and butter samples (Table 2). Because sour cream and butter possess rich bacterial resources and bacterial community diversity, the co-fermentation of all the strains present in the food products may lead to the metabolic differences between the sour cream and butter samples.

To further understand the correlation relationships between the most prevalent bacterial species and the main metabolites, correlation analysis was performed between the most prevalent bacterial species (average relative abundance of >0.5%) and 19 main metabolites in sour cream. The predominant species, *L*. *lactis*, was positively correlated with the 12 metabolites with higher relative abundances, including decanoic acid, palmitic acid, L-xylulose, isohexanal, and some short chain polypeptides, and significantly negatively correlated with some amino acids. Decanoic acid and palmitic acid have been detected in metabolic profiling of *L. lactis* subsp. *cremoris* MG1363 under different culture conditions (Azizan et al., 2012). Previous study on metabolic fingerprinting of bacterial metabolism in a model cheese also demonstrated some amino acids or volatile metabolites can be easily linked to *L. lactis* metabolism (Le Boucher et al., 2013). *L. lactis* has many nutritional requirements, including amino acid, which was the reason of some free amino acids negative with *L. lactis*. The results revealed that prevalent bacterial species were correlated with the main metabolites. However, detailed explanation of the correlation relationship between bacterial species and metabolites in sour cream should perform metagenomic sequencing and metabolic pathway analysis in future study.

We analyzed the bacterial communities and metabolic character of Russian sour cream and butter using a combination of SMRT sequencing techniques and UPLC–Q–TOF mass spectrometry. This study clearly showed that the bacterial community of sour cream and butter are diverse and abundant, and the bacterial compositions have differences between sour cream and butter samples. Meanwhile, a total of 27,822 metabolites were detected in all samples, and 27 significantly different metabolites were detected between the sour cream and butter samples, including short peptides, organic acids, and amino acids. Based on correlation analyses between the most prevalent bacterial species and the main metabolites in sour cream, we conclude that there may be a connection between the dominant LAB species and these metabolites. In addition, 48 strains were isolated by pure culture method and they were identified into seven genera and 15 species and/or subspecies. Those LAB isolates could be used to screen probiotic strains and design starter culture. In conclusion, this study combined omics techniques to analyze the bacterial diversity and metabolic character of traditional sour cream and butter, and we hope that our findings provide valuable information for industrial production of traditional fermented cream.

## Author Contributions

WL and HZ designed the experiments. JY, XA, LM, and CY performed the experiments. LP and DR analyzed the data. JY and TT drafted the manuscript. All authors read and approved the final manuscript.

## Conflict of Interest Statement

The authors declare that the research was conducted in the absence of any commercial or financial relationships that could be construed as a potential conflict of interest.

## Footnotes

1 http://www.megasoftware.net

2 https://www.r-project.org/

3 http://www.hmdb.ca/

4 www.genome.jp/kegg

5 www.chemspider.com

6 http://www.metlin.scripps.edu/

7 http://metagenomics.anl.gov/

## Supplementary Material

FIGURE S1 Relative abundances and diversity of bacteria in the sour cream and butter samples at the genus **(A)** and species **(B)** level.

TABLE S1 All species content of butter and sour cream samples.
