# Supplementary figures and images for: Bacterial Microbiota and Metabolic Character of Traditional Sour Cream and Butter in Buryatia, Russia
Source: Front Microbiol. 2018 Oct 22;9:2496. doi: 10.3389/fmicb.2018.02496 (PMC6232932; doi:10.3389/fmicb.2018.02496)

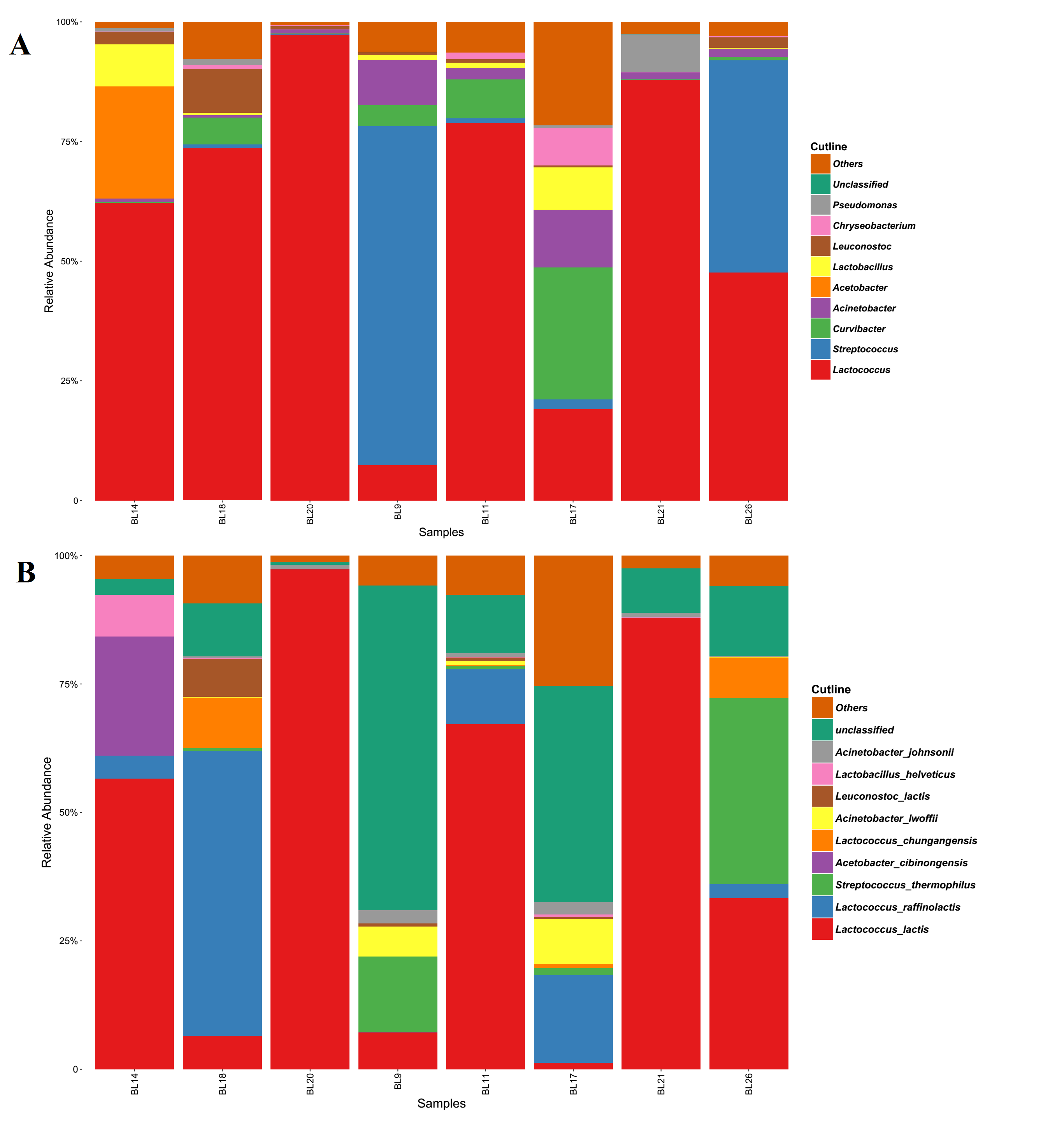

Supplement: FIGURE S1 — Relative abundances and diversity of bacteria in the sour cream and butter samples at the genus (A) and species (B) level. [file Image_1.TIF]
